# Supplementary material for: Identification of lubricant viscosity to minimize the frictional impact of colonoscopy on colonic mucosa
Source: Commun Eng. 2024 Feb 16;3:31. doi: 10.1038/s44172-024-00177-5 (PMC10955975; doi:10.1038/s44172-024-00177-5)
Supplement: Supplementary file 2 — Supplementary Information [file 44172_2024_177_MOESM2_ESM.pdf]

Supplementary Information

Identification of lubricant viscosity to minimize the frictional impact of colonoscopy on colonic mucosa

Naoto Watanabe, Ryohei Hirose , Hiroshi Ikegaya, Katsuma Yamauchi, Hajime Miyazaki, Takuma Yoshida, Risa Bandou, Ken Inoue, Osamu Dohi, Naohisa Yoshida, Takaaki Nakaya, Yoshito Itoh

Correspondence to: ryo-hiro@koto.kpu-m.ac.jp (Ryohei Hirose)

Table of Contents

1. Supplementary Figure S1.
2. Supplementary Figure S2.
3. Supplementary Table S1.
4. Supplementary Table S2.
5. Supplementary Table S3.

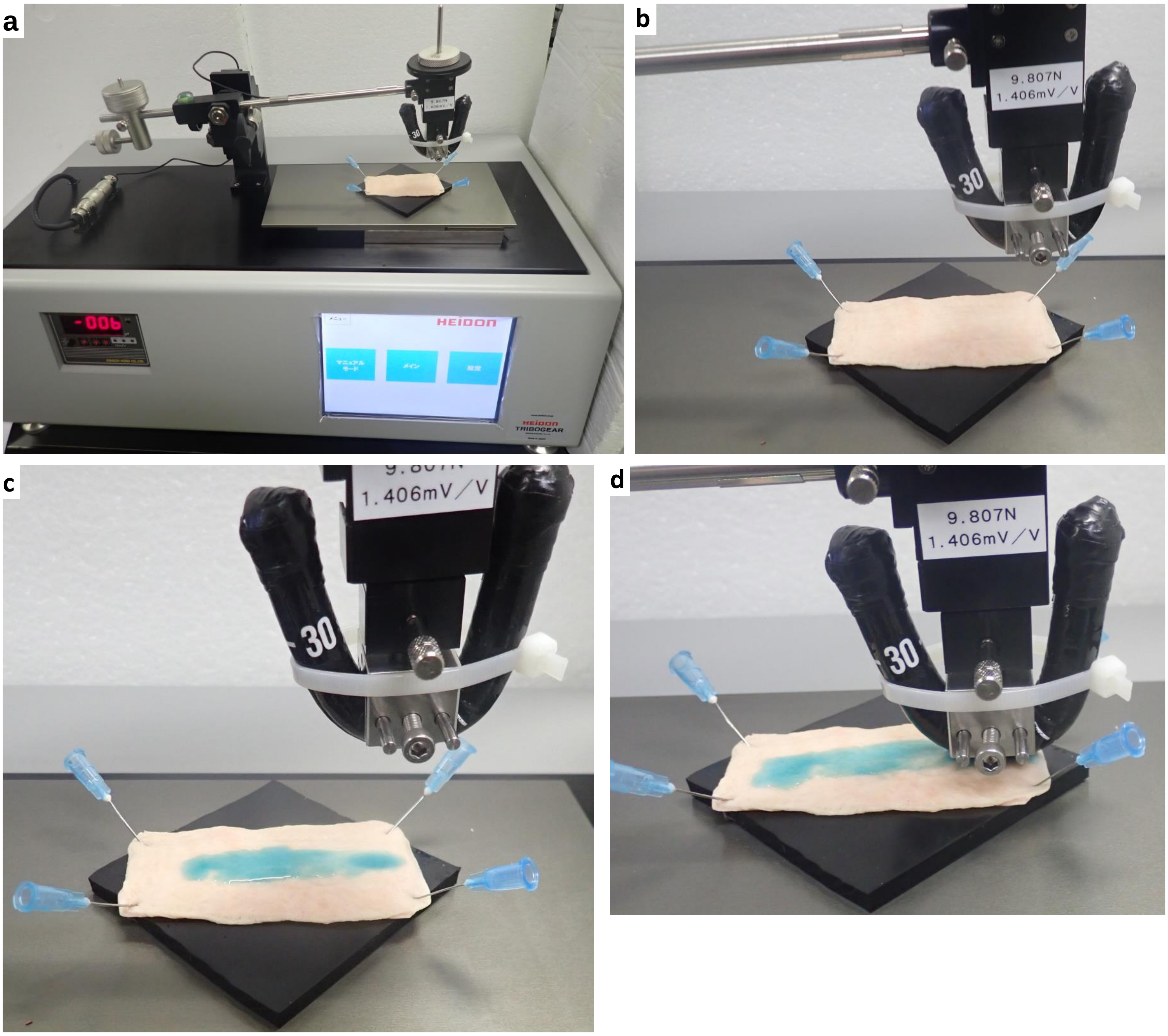

Supplementary Figure S1. Model for measuring DFC between colonic mucosa and endoscopic shaft. The DFC was measured using TRIBOGEAR TYPE 38 and calculated using Tribosoft ver. 6.26 (a). A colon autopsy specimen was fixed to a moving table with a certain tension applied, and the DFC between the colonic mucosa and the endoscopic shaft was measured by attaching the endoscopic shaft portion to the measurement fixture (b). After 0.5 mL of lubricant was evenly applied to the mucosa of the colon autopsy specimen (c), the endoscopic shaft portion was brought into contact with the colonic mucosa and loaded with a 100-g weight. The moving table was set to move a distance of 50 mm at a speed of 1.0 cm/s and a reciprocation frequency of 10 (d). The average of the DFC values measured during the 10 round trips of the moving table was used as the measurement value.

Note: The model was constructed using a porcine colon instead of a human colon because the model is published as a photograph. The lubricant is colored blue for clarity.

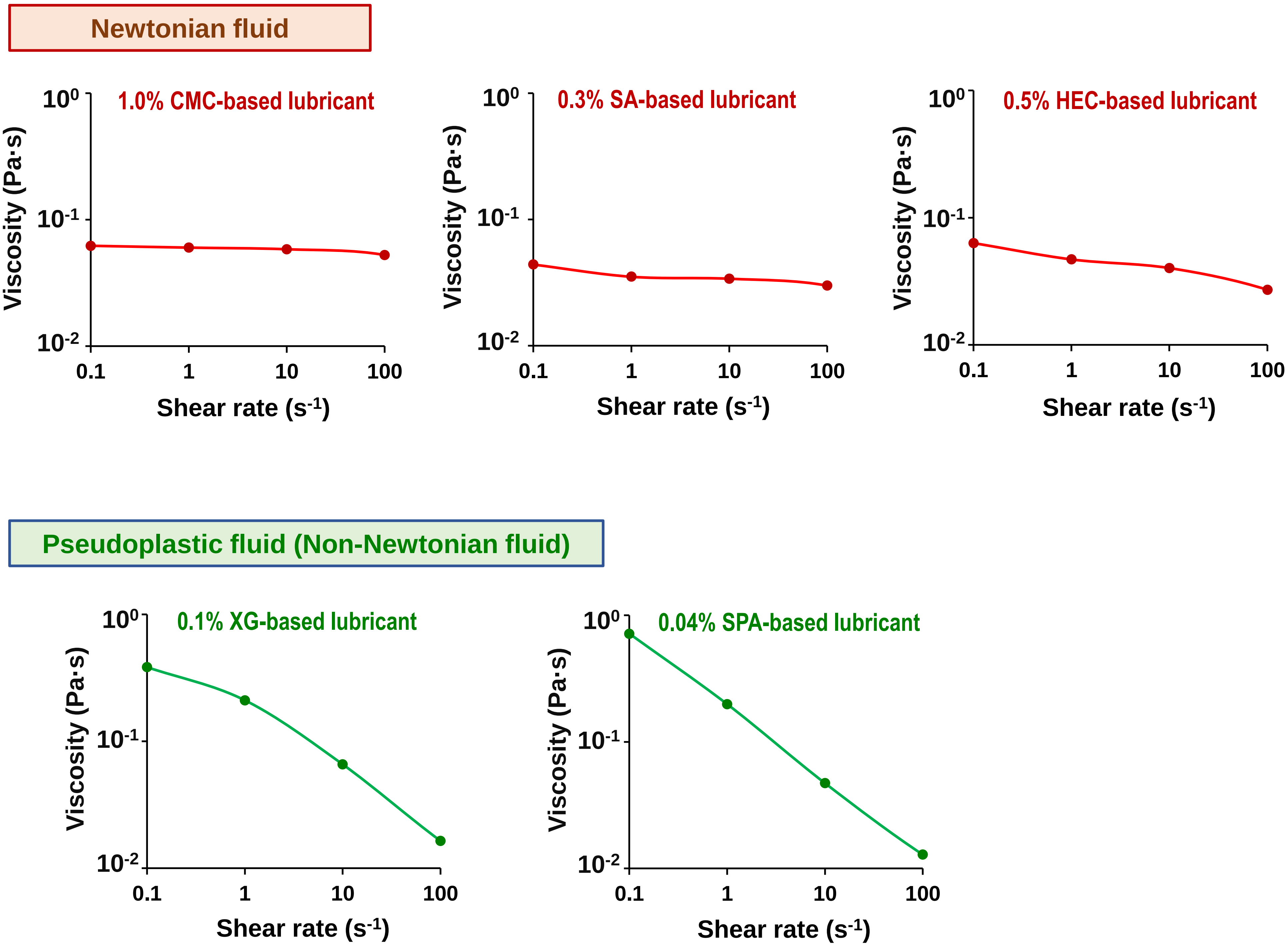

Supplementary Figure S2. Difference in rheological characteristics between a Newtonian fluid and a pseudoplastic fluid (non-Newtonian fluid). The viscosity of a Newtonian fluid remains constant even if the shear rate changes. In contrast, the viscosity of a pseudoplastic fluid varies significantly with changes in the shear rate.

Supplementary table S1. Viscosities of all lubricants.

	Viscosity, mPa･S			
	1 s^{-1}	10 s^{-1}	100 s^{-1}	1000 s^{-1}
**0.01% SA**	8.08	3.33	2.66	1.93
**0.02% SA**	12.31	6.08	4.93	3.06
**0.04% SA**	10.15	8.27	7.31	4.39
**0.05% SA**	33.40	10.64	7.69	4.79
**0.06% SA**	16.11	10.09	8.74	5.47
**0.07% SA**	13.12	10.33	9.30	5.68
**0.08% SA**	23.96	12.39	10.79	6.75
**0.09% SA**	17.32	12.48	11.20	7.19
**0.1% SA**	15.79	12.80	11.63	7.45
**0.2% SA**	21.76	20.48	18.50	11.78
**0.3% SA**	35.24	34.00	29.95	17.49
**0.4% SA**	55.70	52.93	44.94	24.22
**0.5% SA**	80.27	77.92	63.21	31.56
**0.75% SA**	221.63	205.40	141.24	56.57
**1.0% SA**	580.92	516.38	301.87	92.76

	Viscosity, mPa･S			
	1 s^{-1}	10 s^{-1}	100 s^{-1}	1000 s^{-1}
**0.006% SPA**	11.22	5.96	2.98	1.81
**0.007% SPA**	16.12	7.49	3.40	1.94
**0.008% SPA**	19.77	8.42	3.59	1.96
**0.009% SPA**	29.83	10.66	4.12	2.10
**0.01% SPA**	28.71	11.27	4.70	2.49
**0.02% SPA**	98.91	27.76	8.79	3.52
**0.03% SPA**	154.17	39.80	11.55	4.28
**0.04% SPA**	198.65	47.12	12.83	4.44
**0.05% SPA**	260.63	58.88	14.97	4.97
**0.06% SPA**	275.74	65.88	17.33	5.48
**0.07% SPA**	457.61	103.72	24.55	7.30
**0.08% SPA**	557.09	131.63	31.59	9.01
**0.09% SPA**	679.79	154.76	35.62	9.82
**0.1% SPA**	709.54	163.41	37.60	10.28
**0.2% SPA**	1809.24	341.37	69.17	17.80

	Viscosity, mPa･S			
	1 s^{-1}	10 s^{-1}	100 s^{-1}	1000 s^{-1}
**0.1% HEC**	3.24	2.57	2.33	2.11
**0.15% HEC**	5.68	3.75	3.52	2.95
**0.2% HEC**	11.05	6.36	5.41	4.41
**0.25% HEC**	13.70	8.24	7.12	5.03
**0.3% HEC**	16.49	13.23	10.90	7.04
**0.35% HEC**	22.80	17.05	13.54	8.28
**0.4% HEC**	35.40	29.65	21.63	11.60
**0.45% HEC**	39.30	31.41	22.45	11.77
**0.5% HEC**	46.94	40.12	27.09	13.32
**0.6% HEC**	78.42	63.91	38.26	16.61
**0.7% HEC**	114.66	94.91	52.81	20.96
**0.8% HEC**	192.16	139.70	67.33	24.28
**0.9% HEC**	312.37	196.12	86.05	28.56
**1.0% HEC**	455.84	282.39	114.64	35.32
**2.0% HEC**	8237.32	3047.70	800.76	173.23

	Viscosity, mPa･S			
	1 s^{-1}	10 s^{-1}	100 s^{-1}	1000 s^{-1}
**0.01% CMC**	6.56	2.15	1.68	1.50
**0.02% CMC**	4.12	2.42	2.13	1.78
**0.04% CMC**	12.44	3.93	3.04	2.63
**0.06% CMC**	11.13	4.46	3.85	3.13
**0.08% CMC**	9.29	4.83	4.40	3.55
**0.1% CMC**	7.01	5.63	5.46	4.43
**0.2% CMC**	22.78	7.84	6.64	5.50
**0.3% CMC**	16.05	10.70	10.19	7.94
**0.4% CMC**	14.81	13.65	13.27	10.06
**0.5% CMC**	18.64	16.98	16.39	12.19
**0.6% CMC**	22.84	21.95	21.11	15.11
**0.7% CMC**	27.39	26.90	25.67	17.89
**0.8% CMC**	36.71	35.58	33.33	22.05
**0.9% CMC**	47.99	46.72	42.66	27.05
**1.0% CMC**	60.14	58.40	52.56	31.32
**2.0% CMC**	498.04	404.87	260.66	113.89

	Viscosity, mPa･S			
	1 s^{-1}	10 s^{-1}	100 s^{-1}	1000 s^{-1}
**0.01% XG**	22.45	7.12	2.78	1.63
**0.02% XG**	14.40	8.61	4.01	2.08
**0.03% XG**	37.59	15.28	5.47	2.41
**0.04% XG**	59.24	21.97	6.98	2.75
**0.05% XG**	86.00	29.55	8.56	3.09
**0.06% XG**	97.10	31.96	9.30	3.37
**0.08% XG**	177.01	54.33	13.65	4.22
**0.1% XG**	210.22	65.72	16.40	4.78
**0.2% XG**	516.91	135.88	29.42	7.41
**0.3% XG**	938.23	215.28	42.81	10.07
**0.4% XG**	1351.01	286.53	53.86	12.05
**0.5% XG**	1606.44	319.02	58.54	12.96
**1.0% XG**	2683.03	501.88	97.77	22.39

	Viscosity, mPa・S			
	1 s^{-1}	10 s^{-1}	100 s^{-1}	1000 s^{-1}
**Xylocaine Jelly**	3197.9	2285.1	1286.0	432.6
**CaineZero Jelly**	23602.0	7239.3	1485.2	251.2
**Through Projelly**	34845.0	7869.7	1377.1	221.6
**Null Jelly**	47523.0	11909.0	2205.0	363.3
**KY Jelly**	43172.0	12247.0	2369.1	375.0
**Endolubri L**	24567.0	7009.3	1387.5	238.7
**Endolubri H**	46735.0	11738.0	2149.8	347.9

CMC, carboxymethyl cellulose; SA, sodium alginate; SPA, sodium polyacrylate; XG, xanthan gum; HEC, hydroxyethyl cellulose

Supplementary table S2. DFC between colon mucosa and endoscopic shaft.

	Dynamic friction coefficient, mean ± SE
0.01% SA	0.128 ± 0.022
0.02% SA	0.120 ± 0.023
0.04% SA	0.113 ± 0.027
0.05% SA	0.108 ± 0.029
0.06% SA	0.105 ± 0.029
0.07% SA	0.104 ± 0.031
0.08% SA	0.102 ± 0.030
0.09% SA	0.100 ± 0.029
0.1% SA	0.098 ± 0.029
0.2% SA	0.096 ± 0.030
0.3% SA	0.095 ± 0.032
0.4% SA	0.093 ± 0.029
0.5% SA	0.092 ± 0.030
0.75% SA	0.094 ± 0.028
1.0% SA	0.097 ± 0.028

	Dynamic friction coefficient, mean ± SE
0.006% SPA	0.145 ± 0.039
0.007% SPA	0.133 ± 0.042
0.008% SPA	0.128 ± 0.044
0.009% SPA	0.125 ± 0.045
0.01% SPA	0.122 ± 0.043
0.02% SPA	0.112 ± 0.040
0.03% SPA	0.104 ± 0.033
0.04% SPA	0.086 ± 0.013
0.05% SPA	0.087 ± 0.015
0.06% SPA	0.086 ± 0.015
0.07% SPA	0.086 ± 0.015
0.08% SPA	0.085 ± 0.013
0.09% SPA	0.086 ± 0.014
0.1% SPA	0.087 ± 0.014
0.2% SPA	0.087 ± 0.013

	Dynamic friction coefficient, mean ± SE
0.1% HEC	0.111 ± 0.016
0.15% HEC	0.105 ± 0.021
0.2% HEC	0.100 ± 0.024
0.25% HEC	0.098 ± 0.025
0.3% HEC	0.094 ± 0.023
0.35% HEC	0.093 ± 0.024
0.4% HEC	0.091 ± 0.023
0.45% HEC	0.088 ± 0.022
0.5% HEC	0.087 ± 0.022
0.6% HEC	0.087 ± 0.021
0.7% HEC	0.086 ± 0.018
0.8% HEC	0.084 ± 0.016
0.9% HEC	0.083 ± 0.013
1.0% HEC	0.085 ± 0.012
2.0% HEC	0.097 ± 0.012

	Dynamic friction coefficient, mean ± SE
0.01% CMC	0.142 ± 0.035
0.02% CMC	0.135 ± 0.038
0.04% CMC	0.129 ± 0.035
0.06% CMC	0.125 ± 0.036
0.08% CMC	0.122 ± 0.034
0.1% CMC	0.118 ± 0.032
0.2% CMC	0.115 ± 0.030
0.3% CMC	0.110 ± 0.030
0.4% CMC	0.107 ± 0.029
0.5% CMC	0.103 ± 0.029
0.6% CMC	0.102 ± 0.027
0.7% CMC	0.099 ± 0.025
0.8% CMC	0.100 ± 0.026
0.9% CMC	0.098 ± 0.023
1.0% CMC	0.097 ± 0.021
2.0% CMC	0.101 ± 0.021

	Dynamic friction coefficient, mean ± SE
0.01% XG	0.124 ± 0.032
0.02% XG	0.114 ± 0.033
0.03% XG	0.109 ± 0.032
0.04% XG	0.107 ± 0.033
0.05% XG	0.104 ± 0.031
0.06% XG	0.103 ± 0.030
0.08% XG	0.098 ± 0.029
0.1% XG	0.095 ± 0.027
0.2% XG	0.091 ± 0.025
0.3% XG	0.086 ± 0.022
0.4% XG	0.084 ± 0.022
0.5% XG	0.081 ± 0.020
1.0% XG	0.084 ± 0.022

	Dynamic friction coefficient, mean ± SE
water	0.193 ± 0.025
Xylocaine Jelly	0.131 ± 0.004
CaineZero Jelly	0.149 ± 0.010
Through Projelly	0.149 ± 0.012
Null Jelly	0.178 ± 0.012
KY Jelly	0.162 ± 0.001
Endolubri L	0.148 ± 0.007
Endolubri H	0.175 ± 0.011

CMC, carboxymethyl cellulose; SA, sodium alginate; SPA, sodium polyacrylate; XG, xanthan gum; HEC, hydroxyethyl cellulose

Supplementary table S3. DFC between skin surface and endoscopic shaft.

	Dynamic friction coefficient, mean ± SE
**Water**	0.810 ± 0.030
**1.0% CMC**	0.396 ± 0.022
**0.5% SA**	0.590 ± 0.011
**0.08% SPA**	0.406 ± 0.036
**0.5% XG**	0.539 ± 0.050
**0.9% HEC**	0.478 ± 0.010
**Xylocaine Jelly**	0.104 ± 0.001
**CaineZero Jelly**	0.121 ± 0.001
**Through Projelly**	0.119 ± 0.003
**Null Jelly**	0.145 ± 0.003
**KY Jelly**	0.131 ± 0.001
**Endolubri L**	0.123 ± 0.003
**Endolubri H**	0.138 ± 0.002

CMC, carboxymethyl cellulose; SA, sodium alginate; SPA, sodium polyacrylate; XG, xanthan gum; HEC, hydroxyethyl cellulose
